# Quantitative Assessment of Volatile Profile and Sensory Perception of Artisan Bread Made in the City of Valencia

**DOI:** 10.3390/foods13233872

**Published:** 2024-11-29

**Authors:** Gemma Sanmartín, Isabel Elena Sánchez-Adriá, Ana Salvador, Jose A. Prieto, Francisco Estruch, Francisca Randez-Gil

**Affiliations:** 1 Department of Biotechnology, Instituto de Agroquímica y Tecnología de los Alimentos, Consejo Superior de Investigaciones Científicas, Avda. Agustín Escardino, 7, 46980 Paterna, Valencia, Spain; gemma.sanmartin@iata.csic.es (G.S.); i.s.adria@iata.csic.es (I.E.S.-A.); prieto@iata.csic.es (J.A.P.); 2 Department of Physical and Sensory Properties of Food and Consumer Science, Instituto de Agroquímica y Tecnología de los Alimentos (IATA-CSIC), Avda. Agustín Escardino, 7, 46980 Paterna, Valencia, Spain; asalvador@iata.csic.es; 3 Department of Biochemistry and Molecular Biology, Universitat de València, Dr. Moliner 50, 46100 Burjassot, Valencia, Spain; francisco.estruch@uv.es

**Keywords:** bread dough, biochemical parameters, local bread, VOCs, sensorial evaluation

## Abstract

Artisan bread, known for its simple recipes, natural ingredients, and traditional techniques, has seen a surge in demand, especially following the COVID-19 pandemic. Small bakeries emphasize extended fermentation processes and prioritize sensory qualities in their products. However, the impact of ingredients on the quality characteristics of artisan bread remains underexplored. Here, a set of breads from artisanal bakeries in Valencia, Spain, was characterized. Bread dough pH, total titratable acidity (TTA), and acid content were influenced by flour type and sourdough use, creating different environments for volatile compound (VOC) generation. Over 50 VOCs, including aldehydes, alcohols, acids, and furans, were identified in crumb and crust samples of most artisan bread samples, compared to fewer than 20 VOCs in control industrial bread, where ketones dominated. Whole flours, such as spelt, durum wheat, or T80, along with the leavening agent, affected the abundance of certain volatiles, particularly in the crust. Additionally, the use of spelt or wheat flour impacted crumb texture, while sourdough improved taste intensity, acidity, and crumb color. Finally, certain sensory attributes were also influenced by the presence of hydrocarbons and furans in the volatile fraction of both crumb and crust. Overall, the results provide new insights into the influence of ingredients on the quality of artisan bread and can help bakers refine recipes while maintaining a natural ingredient list. Hence, the work is substantial for the artisan bread industry and consumers.

## 1. Introduction

In Mediterranean countries, artisan bread is a vital part of the culture, as it is generally characterized by the use of old recipes and associated with specific locations [1]. Like other artisan foods, the artisan bread way is a combination of natural ingredients and traditional methods, often handmade or not fully mechanized, and produced in limited quantities [2]. Concerning the product, small bakers focus on long fermentations and have flavor and aroma as essential elements of artisan bread [3]. These characteristics together with a trend toward valuing quality and authenticity have boosted a change in consumption choices that have increased the demand for these products. For example, in southern Italy, the purchases of artisanal bread currently account for about 70% of total sales, a figure that increased in particular after the outbreak of COVID-19 [4]. Indeed, during the pandemic, consumers became more health-conscious and preferred products perceived as fresher, more natural, and healthier. All of these factors, combined with a greater sense of community and support for local economies, drew consumers away from industrial bread [4]. Nevertheless, artisan bakers face numerous risks and challenges that jeopardize their existence. For example, in some countries there is no regulation about the use of the term “artisan”, which has allowed the appearance of mass-produced traditional breads [2]. As an alternative, artisan bakers can develop strategies to mitigate industrial competence and meet consumer demands. These should be based on a wider understanding of why bakers are doing what they’re doing, and, in particular, how ingredients and traditional methods influence bread quality.

Bread aroma is considered an essential quality feature and a determinant factor in a consumer’s purchase decision [5]. Volatile compounds in bread include almost all chemical families, with alcohols as the most represented category, followed by esters, ketones, aldehydes and acids [6,7]. Among individual compounds, (E)-2-nonanal (green, tallow), 3-methyl1-butanol (balsamic, alcoholic), hexanal (green), and 2,3-butanedione appear as the most aroma-active contributors [8] from a total of most than 300–400 molecules identified [9]. They originate mainly from the metabolic activity of yeast and lactic acid bacteria, from oxidation of flour lipids during mixing and from Maillard reactions on the dough surface [10]. Concerning microbial activity, the role of the sourdough microbiota in generating volatile compounds from the catabolism of sugars and amino acids is well-established [8,11,12,13,14]. Likewise, differences in operating conditions, including the number of dough refreshments, or time and temperature adopted in the proofing have been identified as influencing sensorial characteristics of bread [15]. However, the impact of ingredients on bread aroma and sensory evaluation has been less investigated.

Flour has always been considered a crucial source of volatile precursors [16]. Indeed, different wheat [17] and spelt [18] varieties lead to different aroma profiles in their respective breads. However, additional factors, such as the environment in which the variety is cultivated [17] and the interactions between different types of flour and yeast, appear to influence bread aroma to a similar extent [15]. Salt also plays an important role in bread flavor due to its effects on microbial activity and physicochemical properties [19], although contradictory results have been reported [20,21]. Additionally, aroma perception is complex, as combined volatiles yield different flavors than those expected from individual molecules, in a matrix-dependent manner [22]. Thus, understanding how all these factors influence aroma formation remains a challenge that needs to be addressed.

Here, we characterized a collection of breads from artisanal bakeries in the city of Valencia, Spain, representing a wide variety of recipes and traditional methods of artisan breadmaking. The objective of this study was to determine the impact of ingredients on the biochemical properties of dough and the volatile profile of bread, as well as how these factors influence sensory perception. We believe our data provide new insights into how these elements affect aroma formation and will equip bakers with the skills to experiment with novel flavor profiles based on consumer preferences and trends. Additionally, this research can guide the use of alternative or local ingredients, offering insights for creating high-quality products in different regions.

## 2. Materials and Methods

### 2.1. Bakeries and Samples Collection

Sixteen types of artisan bread and their respective doughs from twelve bakeries affiliated with the Bakers and Pastry Artisan Trade Union of Valencia (http://www.el-gremio.org, accessed on 15 January 2024) were analyzed in this study (Table 1). Additionally, a bread sample purchased from a local supermarket was used as a control for industrially prepared bread in certain experiments, as dough samples for this control were unavailable. In each bakery, samples were collected, refrigerated, and transported to the laboratory under controlled conditions. Upon arrival, bread dough samples were frozen at −20 °C for further characterization, while bread samples were separated into crumb and crust, cut into squares, and frozen at −80 °C for at least 24 h before volatile compound (VOC) analysis. At least three independent loaves of each type of bread were analyzed.

### 2.2. Determination of pH, TTA, and Organic Acids

Water extracts of bread dough samples were prepared by blending 20 g of dough with 200 mL of sterile distilled water for 3 min in a BagMixer 400 blender (Interscience, Saint Nom la Bretêche, France). pH value was measured with a portable pH-Meter 98190 (HANNA Instruments, Spain), and a portion of the extract was centrifuged (4000 rpm, 15 min, 4 °C) and used to determine the total titratable acidity (TTA). For this, 25 mL of clear supernatant (in triplicate) were titrated using the automatic potentiometric titrator Excellence T5 (Mettler Toledo, Columbus, OH, USA), and the results were expressed as the amount (ml) of 0.1 N NaOH required to reach a pH of 8.5. Lactic and acetic acids determination was carried out by HPLC according to [23]. Briefly, aliquots (10 mL) of homogenized dough were first mixed with 5 mL of 1 mM HClO_4_, and centrifuged at 4000 rpm, 15 min, 4 °C. Then, the clear supernatant was adjusted to pH 3.0, placed on ice for 30 min and centrifuged at 13,000 rpm for 5 min. Finally, the extracts were filtered through 0.45-μm hydrophilic PTFE filters and 20 μL were injected into a C18 3 μm Atlantis T3 column (100 Å, 4.6 × 150 mm; Waters, Milford, MA, USA). Chromatographic separation was conducted in isocratic conditions with a 1 mL/min flow rate of 0.5 M HClO_4_ at 35 °C. Run time, including separation and column washing, was 30 min. Samples were run in triplicate and values are expressed as the mean (±SD).

### 2.3. Volatile Compounds Analysis

The volatile organic compounds (VOCs) were analyzed by headspace solid-phase micro-extraction technique (HS-SPME) and gas chromatography–mass spectrometry (GC–MS) according to [14] with some modifications. Briefly, for each analysis, 0.2 g of crust or crumb bread was placed into a 20 mL headspace vial. Then, 1 mL of milli-Q water and 5 µL of internal standard solution (42 µg/mL, 2-heptanone, Sigma, San Luis, MO, USA) were added to the vial. The headspace was sampled by inserting a 95 μm Carboxen/Polydimethylsiloxane (CAR/PDMS) fiber (Agilent Technologies, Santa Clara, CA, USA). Using an MPS robotic autosampler (Gerstel, Mülheim an der Ruhr, Germany), the vial, previously sealed with a silicon septum, was then heated in an oven at 50 °C for 42 min and the SPME fiber was exposed during the last 30 min. When the extraction time expired, the fiber was immediately inserted into the injection port of an Agilent 7890B-5977B GC/MSD system equipped with an HP-5 capillary column (30 m × 0.25 mm × 0.25 μm) (Agilent Technologies) for the desorption of compounds. The gas carrier was helium with a flow rate of 0.9 mL/min, and SPME injections were placed in the splitless mode at 250 °C for 3 min. The temperature program was set to 35 °C for 3 min, then increased to 160 °C at a rate of 4 °C/min, followed by an increase to 240 °C at a rate of 10 °C/min, held for 2 min, and then held post run at 250 °C for 2 min. The temperatures of the injection port, ion source and transfer line were 250, 230 and 260 °C, respectively. To detect all the compounds, the scan mode in the m/z range of 29–400 amu (atomic mass unit) was used with an electronic impact of 70 eV. Volatile compounds were identified and confirmed by the comparison of the mass spectral data obtained with those in the NIST (National Institute of Standards and Technology) library. The abundance of each volatile was calculated as % of total pick normalized areas, which were obtained after dividing the peak area of the identified compounds by the peak area of the internal standard. At least three independent samples analyzed in triplicate were conducted.

### 2.4. Descriptive Analysis

#### 2.4.1. Selection of Terms

Conventional profiling—QDA^®^ was carried out using quantitative descriptive analysis methodology [24]. A panel of ten assessors, aged between 30 and 65 years and familiar with bread making, was trained to select the descriptors using the checklist method [25]. The sensory test was conducted in a standardized sensory evaluation room with 10 separate booths.

The development of the sensory profile consisted of two sessions. Attributes were selected and discussed in an open session with the panel leader, and a unique list of attributes was established by consensus. The assessors were first given a brief outline of the procedures, a list of attributes, and representative samples. They were then asked to choose and write down the most appropriate attributes to describe all sensory properties of the bread or to suggest new ones. The panel leader collected all the attributes and wrote them on a board, and the panel discussed the appropriateness of the selected attributes, their definitions, and preliminary methods for assessing the products. By the end of this session, a consensus on the list of attributes was reached: crust thickness, crumb cell number, crumb cell size, crumb color, tender texture, fluffy crumb, compact crumb, acid taste, and taste intensity.

#### 2.4.2. Panel Training

The training involved ten sessions, during which each attribute was presented to the panelists along with definitions and physical references to facilitate their understanding. In each session, panelists tasted different samples to achieve a better understanding and final consensus on how to evaluate all descriptors. Tastings continued until the panel’s assessments were homogeneous. Following this, the panelists used a 10 cm unstructured scale to score the selected attributes of the breads.

#### 2.4.3. Formal Assessment

Sample evaluation was carried out in duplicate across eight sessions, with four to five breads evaluated in each session to cover all samples. A balanced complete block experimental design was used to evaluate the samples. The intensities of the sensory attributes were scored on a 10 cm unstructured line scale. The samples were served in random order, each on a separate plastic tray labeled with a random three-digit code. All participants were required to read and sign an informed consent form before the start of the test. Panelists were instructed to rinse their mouths with water between each sample evaluation to minimize interference.

### 2.5. Statistical Analysis

Analysis of variance (one-way ANOVA) was applied to the VOCs analysis to study the influence of sourdough on the aroma of bread. The least significant differences were calculated by Tukey’s test and the significance at *p* < 0.05 was determined. The principal component analyses (PCA) of the instrumental analysis scores and mean scores in the sensory tests were plotted using XLSTAT software (XLSTAT 2018, Addinsoft, Barcelona, Spain). The normalization of the VOC data and the correlation matrix was carried out using MetaboAnalyst [26] (https://www.metaboanalyst.ca/, accessed on 18 September 2024).

## 3. Results and Discussion

### 3.1. Recipes of Artisan Breads

Table 1 displays the local names of the artisan bread evaluated, along with the bakery where they were made and the primary ingredients used in their preparation by the Artisan Association of Valencia City. Except for the ‘Pascua’ bread (G4), an enriched Easter bread, and the specialties (G7 and G11), the other bread were characterized by minimalist recipes, consisting of just a few ingredients—typically only the basic components of bread: flour, water, yeast, and salt—with most cases showing an absence of additives (only 2 out of 16), all indicating artisanal rather than industrial processing conditions [27].

The formulations were further distinguished by incorporating various flour blends (10/16), such as durum wheat, high-protein wheat, stoneground white, whole rye, or spelt (as listed in Table 1). This approach aimed to introduce product diversity and maintain high finished-product quality without relying on dough conditioners. The use of sourdough to enhance flavor, aroma, and open crumb textures, among other qualities [28,29,30,31], and to reduce (G1, G3_F, G3_DW, G4, G10) or avoid (G3_SDw, G9_L, G12) the use of commercial yeast for dough leavening was also very popular (13/16). Moreover, the addition of fat in the bread collection was uncommon (2/16), with vegetable oil being the characteristic ingredient rather than butter (Table 1). Finally, the set of artisanal breads analyzed was characterized by long fermentation periods at different temperatures often combined with at least one retarding time (Table S1). Thus, our bread samples appear to align well with existing definitions of artisanal food [2]. Additionally, they are produced in limited quantities, in small bakeries, and within the local area of Valencia City.

### 3.2. Biochemical Characterization of Bread Dough

We tested the main biochemical parameters of bread dough samples corresponding to the artisan bread analyzed (Table 2). The pH values ranged from 3.76 ± 0.08 to 5.58 ± 0.02, while TTA values ranged from 9.8 ± 0.3 to 2.30 ± 0.08 mL of 0.1 N NaOH (Table 2). Lactic acid content was consistent with the TTA data, ranging from 0.19 ± 0.02 to 11.2 ± 1.0 mg/g of dough (fresh basis, f.b.). The median concentration of acetic acid was 1.13 mg/g of dough (f.b.), with variations from 0.16 ± 0.12 to 3.2 ± 0.7 mg/g (Table 2). Notably, acetic acid levels were highest in the G1, G5_S, and G5_R dough samples. A common belief is that higher levels of acetic acid enhance the sourness of dough and the flavor of bread [32]. A low hydration level (G1), which promotes acetic acid production, together with the use of whole grain flours, such as T80, spelt, or whole rye in G1, G5_S, and G5_R, respectively (Table 1), could account for the high acetic acid content in these dough samples (Table 2). These flours, often referred to as ‘high extraction’ flours, have a higher ash and nutrient content, which can lead to higher acetic acid levels due to the increased fermentation activity they support [33].

As expected, the use of sourdough contributed to increased acidity and reduced dough pH. Consistent with this, dough samples that did not contain sourdough, such as G7, G11 and G13, exhibited the highest pH and lowest TTA values (Table 2). Additionally, variations in these parameters may reflect the influence of other factors, such as flour type, which primarily determines buffering capacity, and processing conditions [31]. For example, proofing at very low temperatures (7–8 °C; Table S1) could explain pH values around 5.0 in samples like G3_F, G6, and G10, which contain sourdough proportions of 20%, 30%, and 25%, respectively (Table 1).

Regarding the fermentation quotient (FQ, the molar ratio between lactic and acetic acids), a determining factor for the balanced acidic taste of bread [32], values fluctuated from 1.08 to 5.40 in all samples, except for G12 and G13, which had the highest (7.44) and lowest (0.32) values, respectively, among the breads analyzed (Table 2). Bread dough G12 contained a high proportion of sourdough (35%), previously characterized by an exceptional lactic acid bacteria cell count exceeding 9.0 log₁₀ CFU/g [14], while the low FQ in G13 could reflect reduced fermentation activity due to the absence of sourdough and low water content, among other factors (Table 1). In contrast, samples G3_F, G6, and G10, characterized by the highest acetic content (see above), showed FQ values within the optimal range of 2.0–2.7 [34].

Altogether, the sixteen types of artisan bread analyzed represent distinct environments for volatile compound generation, which could significantly influence sensory perception during the tasting.

### 3.3. Volatile Profile of Artisan Bread: From Crumb to Crust

**Figure 1:**
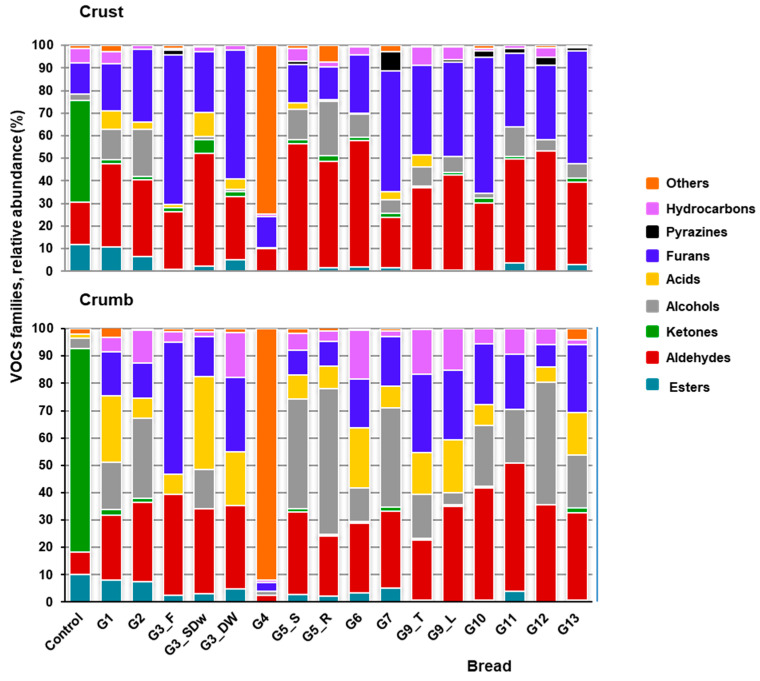
Abundance of volatile families in the crumb and crust of artisan bread. The relative abundance of each volatile chemical family was calculated as the sum of the percentages of the corresponding individual species. Values represent the mean percentage (%) of the total peak normalized area for each family.

Fifty-one and fifty-five volatile compounds were identified in the crumb and crust samples, respectively, of most artisan breads, except for the control industrial bread, in which fewer than twenty were found (Tables S2 and S3). Regarding chemical classes, aldehydes and alcohols, along with acids and furans, were the most abundant categories in bread crumb (Figure 1), with hexanal, 3-methyl-1-butanol, 1-hexanol, phenylethyl alcohol, acetic acid, and 2-pentylfuran as the major molecular species (Table S2). Volatile compounds in bread crumb originate mainly from sugar and amino acid degradation during fermentation, lipid oxidation (e.g., hexanal), and to a lesser extent, dehydration/fragmentation (furans) during the Maillard reactions [8]. In general, they contribute odors of cut grass, citrus and green, having a significant impact on bread aroma [7]. Unlike this aroma profile, fewer than twenty VOCs were detected in the crumb of the control industrial bread, with ketones as the most representative family (Figure 1). Additionally, eight molecular species, including amyl acetate and the ketones 5-methyl-2-hexanone and 2,6-dimethyl-4-heptanone, were present exclusively in this bread (Table S2). This analysis highlights the strong impact of processing methods—artisanal or industrial—likely linked to differences in fermentation conditions and ingredients, particularly sourdough [6], on the aromatic complexity of bread.

These differences were also evident in the volatiles of crust samples, where ketones once again emerged as the primary family in the control industrial bread (refer to Table S3). In contrast, the artisan bread samples exhibited a predominance of compounds formed primarily through heat-induced Maillard reactions, such as hexanal (described as leafy, grassy and green) and heptanal (noted for its citrus, fatty and green notes), along with furans, including furfural (characterized by almond, baked potato, bread, burnt and spice aromas) and 2-pentylfuran (described as buttery, floral, fruity and reminiscent of green beans). These compounds were among the most abundant, while fermentation-induced alcohols and acids decreased in relative abundance (refer to Figure 1 and Table S3). As expected, the increased production of pyrazines on the bread surface was also evident, with two major species identified, namely 2-ethylpyrazine (popcorn, nutty) and 2-ethyl-6-methylpyrazine (potato, roasted), while the level of hydrocarbons decreased (Figure 1; Table S3). Pyrazine formation via Strecker degradation involves α-amino acids and α-dicarbonyl compounds as starting substrates and is favored at temperatures between 120 and 150 °C [35]. The results further emphasized the differences between artisanal and industrial methods, as well as between crumb and crust, in contributing to the intensity and quality of bread aroma.

### 3.4. Influence of Ingredients on Volatile Profile

We evaluated the impact of the ingredients used in the preparation of our set of artisanal breads on the VOCs generated during leavening and baking in both the crumb and crust. Positive and negative correlations were considered based on R^2^ ≥ 0.6 and R^2^ ≤ −0.6, respectively, and *p* < 0.01. As shown in Figure S1, the amount of water used in each recipe had a positive effect on the hexanal content in the crumb. Although aldehydes are primarily derived from the lipid oxidation of unsaturated fatty acids [6], certain aldehydes, such as hexanal, can also be generated by microbial metabolism. In particular, this occurs in sourdough, where heterofermentative LAB such as *F. sanfranciscensis* are the predominant species [14].

In contrast, a negative correlation was found between yeast dosage and 3-ethyl-2-methyl-1,3-hexadiene (Figure S1). The use of certain types of flour also appeared to favor the generation of specific hydrocarbons, such as 1-nitrohexane in tritordeum flour, or had a negative impact on the content of other volatiles, such as heptanal, in the crumb of bread made from durum wheat (Figure S1).

The correlation between VOC abundance in crust samples and the main ingredients in the artisan bread set was also analyzed (Figure S2). In general, Pearson’s plot showed a higher number of correlations in the crust than in the crumb, consistent with the higher baking temperature at the bread surface and the role of baking as a primary factor in determining bread aroma. The analysis also indicated a stronger contribution of whole flours to the individual content of certain alcohols released from the crust. Specifically, spelt flour was positively correlated with 2-methylbutanol, durum wheat with 3-methylbutanol, and T80 flour with 3-decen-1-ol and 5-decen-1-ol. Given that many of these compounds are fermentation volatiles, the results suggest an interaction between microbial metabolic pathways and the type of flour used in each bread recipe.

In addition, the leavening agent also had a significant effect on the aromatic profile of the bread crust. Crust from sourdough-leavened bread displayed higher levels of 3-methylbutanal and heptanal, while the dosage of commercial baker’s yeast again correlated negatively with the level of the hydrocarbon 3-ethyl-2-methyl-1,3-hexadiene. Finally, significant correlations were also found for some minor ingredients: tritordeum flour and oil were associated with 1-nitrohexane and 1-octen-3-ol, respectively (Figure S2).

### 3.5. Sensory Evaluation

**Figure 2:**
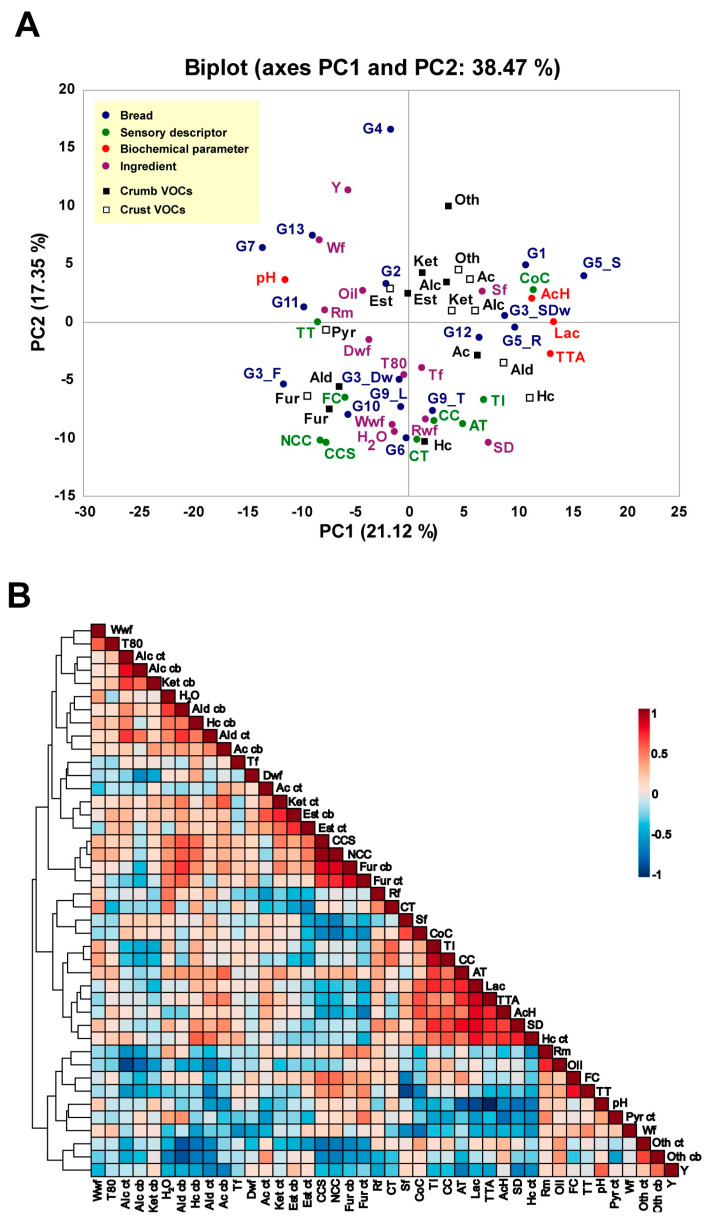
Principal component analysis (PCA) and correlation plot. (**A**) PCA based on values of the sensorial evaluation of nine bread quality descriptors [crust thickness (CT), number of crumb cells (NCC), crumb cells size (CCS), crumb color (CC), tender texture (TT), fluffy crumb (FC), compact crumb (CoC), acidic taste (AT), taste intensity (TI)], biochemical parameters [pH, TTA, acetic and lactic, (Table 2)], abundance of volatile families [esters (Est), alcohols (Alc), ketones (Ket), acids (Ac), furans (Fur), hydrocarbons (Hc), and others (Oth)] in crumb (Table S2) and crust (Table S3), and main ingredients [wheat flour (Wf); whole wheat flour (Wwf); T80 flour (T80); water (H_2_O); sourdough (SD); yeast (Y); whole rye flour (Wrf); roasted malt (Rm); durum wheat flour (Dwf); tritordeum flour (Tf); and spelt flour (Sf)] in the set of artisan breads analyzed (Table 1). (**B**) Pearson’s correlation between pairs of main ingredients, biochemical parameters (Table 2), and abundance of volatile families in crumb (Table S2) and crust (Table S3) samples from the set of artisan breads analyzed (Table 1). Pearson’s correlation index is indicated by the scale bar, with 1 denoting perfect positive correlation (dark red) and -1 denoting perfect negative correlation (dark blue). Abbreviations are as in (**A**).

A sensory evaluation was conducted to enhance the understanding of overall perceived bread quality using nine sensory descriptors and to explore their relationship with volatile compounds, dough biochemical parameters, and ingredient composition. Figure 2A shows the principal component analysis (PCA), which explained 38.47% of the total observed variability. In general, a low percentage of explained variability indicates that the sensory data have complex characteristics and are influenced by additional factors, such as process conditions or ingredient traits that cannot be easily summarized by only a few components [36,37]. Indeed, kneading, leavening, and baking are critical steps for bread quality and strongly influence sensory perception [6,7]). Additional variables, including cereal genotype, gluten composition, and flour type, can also have a significant impact on sensory evaluation. For example, Ficco et al. [38] identified the milling procedure as the largest determinant of bread sensory attributes, particularly for color, smell, and taste, and to a lesser extent for crust and crumb firmness, appearance, and alveolation.

Nevertheless, the results illustrated in Figure 2A show that different facets of bread quality were still associated with the ingredients used and the biochemical properties of dough and bread. PC1 accounted for 21.12% of the variability and was associated with the use of spelt, oil, roasted malt, and durum wheat, as well as acetic acid, lactic acid, TTA, and pH parameters. PC2 explained 17.35% of the variability and was primarily related to the amount of water and the use of wheat, tritordeum, and whole wheat flour, along with most of the sensory descriptors.

Bread samples displaying the lowest dough pHs as G1, G5_S, G3_SDw, G5_R, and G12 (Table 2), appeared on the positive side of PC1 and were linked to the presence of acids, ketones, and the sensory descriptor “compact crumb” (Figure 2A). In general, acidic pHs weaken the gluten network, leading to a decrease in the gas retention capability, which results in a denser crumb and a firmer texture [39]. Meanwhile, samples G2, G7, G11, and G13, all characterized by a high dough pH and low TTA values (Table 2), were associated with esters and pyrazines and the sensory descriptor “soft texture” on the negative side of PC1. Additionally, sample G4 was associated with the presence of yeast on the positive side of PC2, while samples G3_F, G3_DW, G6, G9_L, G9_T, and G10 were linked to sensory descriptors like fluffy crumb, the number and size of crumb cells, crust thickness, crumb color, and acidic taste, along with aldehydes and furans on the negative side of PC2 (Figure 2A).

Finally, the data collection was represented in a heat map (Figure 2B), and the correlation among ingredients, volatile compound abundance, and main dough biochemical parameters was analyzed. Positive and negative correlations were considered based on R^2^ ≥ 0.6 and R^2^ ≤ −0.6, respectively, and *p* < 0.01. Significant correlations were found among different sensory descriptors, ingredients, and biochemical characteristics of bread dough (Figure 2B). As expected, the use of sourdough, and the resulting increases in dough TTA and lactic acid content, positively correlated with acidic taste and taste intensity, while the incorporation of commercial yeast reduced panelists’ perception of acidic taste. Sourdough and lactic acid also positively affected crumb color, while TTA and acetic acid were primarily associated with a compact crumb (Figure 2B).

The use of spelt or wheat flour influenced crumb texture perception, with furans in both crumb and crust positively influencing the number and size of crumb cells. Finally, crumb color was also positively affected by the content of hydrocarbons. No other statistically significant relationships were observed between the volatile family content and the sensory attributes analyzed (Figure 2B). In summary, additional factors, including the interaction between volatile compounds should be considered as potential contributors to the differences in sensory properties perceived in this study.

## 4. Conclusions

Our study on a set of artisan breads produced by the Guild of Bakers and Confectioners of Valencia reveals key ingredients that play a critical role in generating distinct volatile compounds and driving biochemical changes. The type of flour, whether whole or refined, and the leavening agent used—sourdough versus commercial baker’s yeast—significantly affect the total titratable acidity (TTA), as well as the lactic and acetic acid content of the bread dough. These factors, in turn, contribute notably to the panelists’ perception of taste intensity, acidity, crumb color, and texture in the bread samples.

Similarly, the volatile organic compound (VOC) profile of both the crumb and crust is influenced by the composition of the artisan bread, with high-ash flours and leavening agents playing a prominent role in the abundance of major alcohols and aldehydes, including 2-methylbutanol, 3-methylbutanol, 3-methylbutanal, and heptanal. Notably, all artisan breads exhibit a much more complex volatile profile than the control industrial bread, underscoring the strong impact of fermentation time and ingredients on bread aroma. Some sensory attributes, such as crumb color and the number and size of crumb cells, appear to correlate with the content of certain volatiles. However, further research is needed to fully understand how VOCs influence these sensory properties in the final bread.

Our findings emphasize the importance of flavor development through extended fermentations and the use of high-ash flours, both of which positively influence aromatic diversity and fermentation vigor. These results align with previous studies characterizing artisan bread, particularly within the artisan baking community in the United States [3]. Overall, this research can guide baking professionals in experimenting with flour blends, new cereal varieties such as ancient grains, and various sourdoughs to create a higher-quality range of products tailored to meet consumer demands and expectations.

Future research should analyze additional samples and consider other factors influencing artisan bread quality. Particular attention should be given to textural properties, evaluated either instrumentally or sensorially, and their interactions with other sensory attributes and aroma perception/production. These considerations could contribute significantly to a more comprehensive understanding of artisan bread quality.

## Figures and Tables

**Table 1:** Recipes of artisan bread analyzed in this study.

Bread ^1^	Denomination ^2^	Origin	N° Ingredients	Bakery	Ingredients ^3,4^	% (f.b.)
**Control**	*Común* (Standard bread)	Industrial	4	Unknown	Wheat flour	Not available
					Tap water	
					Yeast	
					Table salt	
**G1**	*Huerta*	Artisanal	6	Llàtcer	160 W wheat flour (Harinas Santamaría)	75
					Bio T80 flour (Harinera Roca)	25
					Tap water	62
					Sourdough	20
					Compressed yeast	0.4
					Table salt	1.8
**G2**	*Pueblo*	Artisanal	5	San Pablo	>120 W wheat flour (Panificadora Conquense)	100
					Tap water	75
					Sourdough	20
					Compressed yeast	1.0
					Table salt	1.75
**G3_F**	*Francés* (French baguette)	Artisanal	8	La Tahona del Abuelo	320 W wheat flour (Harinas Vicente Bosque)	80
					Whole rye flour Type 170 (Dossche Mills)	20
					Tap water	85
					Sourdough	20
					Olive oil	5.0
					Table salt	1.8
					Roasted malt (Bayogar)	0.5
					Compressed yeast	0.3
**G3_SDw**	*Masa madre trigo* (Wheat sourdough bread)	Artisanal	4	La Tahona del Abuelo	200 W wheat flour (Harinas Vicente Bosque)	100
					Tap water	70
					Sourdough	20
					Table salt	1.7
**G3_DW**	*Trigo duro* (Durum wheat bread)	Artisanal	5	La Tahona del Abuelo	Durum wheat flour (Harinas Belda)	100
					Tap water	80
					Sourdough	20
					Compressed yeast	0.3
					Table salt	1.8
**G4**	*Pascua* (Easter bread)	Artisanal	9	Marco artesans	90–100 W wheat flour (Harinas Santamaría)	100
					Tap water	56
					Raisins	8.0
					Sourdough	5.0
					Walnuts	4.0
					Montanejos Honey (Casa Bautista)	2.5
					Refined sunflower oil (High oleic, Casalbert)	2.0
					Compressed yeast	4.0
					Table salt	1.8
**G5_S**	*Espelta* (Spelt bread)	Artisanal	6	Valencia	Whole spelt flour (El Molino de Cerecinos)	100
					Tap water	80
					Sourdough	20
					Compressed yeast	0.8
					Table salt	1.7
**G5_R**	*Centeno* (Rye bread)	Artisanal	6	Valencia	Whole rye flour (Harivasa)	60
					180–220 W wheat flour (Harinas Santamaría)	40
					Tap water	80
					Sourdough	20
					Table salt	1.7
					Compressed yeast	0.8
**G6**	*Carrasca*	Artisanal	7	Monpla	180 W wheat flour (Harinas Molí de Picó)	70
					Bio T80 flour (Moulin de Colagne)	10
					Whole wheat flour (Harinera Coromina)	10
					Whole rye flour (Harinas Molí de Picó)	10
					Tap water	85
					Sourdough	30
					Table salt	1.8
**G7**	*Chapata* (Ciabatta bread)	Artisanal	7	Masanet	380–400 W wheat flour (Harinas Saiz)	60
					220–260 W wheat flour (Harinas Saiz)	40
					Tap water	80
					Table salt	1.1
					Compressed yeast	1.8
					Bread improver (T500, Puratos)	0.4
					Roasted malt (Bayogar)	0.1
**G9_T**	*Tritordeum* (Tritordeum bread)	Artisanal	6	La Tahona del Boni	T72 tritordeum flour (Molinos del Duero)	100
					Tap water	75
					Sourdough	20
					Compressed yeast	0.08
					Table salt	1.7
					Seeds mix (Molí de Picó)	1.8
**G9_L**	*Llesca*	Artisanal	5	La Tahona del Boni	200 W wheat flour (Molinos del Duero)	90
					Whole rye flour (Molinos del Duero)	10
					Tap water	75
					Sourdough	20
					Table salt	1.7
**G10**	*Moruno*	Artisanal	7	Vicente Raimundo	200 W wheat flour (Harinas Saiz)	90
					Whole wheat flour (Harinera Segorbina)	5
					Whole rye flour (Harivasa)	5
					Tap water	83
					Sourdough	25
					Table salt	0.2
					Compressed yeast	1.8
**G11**	*Chapata* (Ciabatta bread)	Artisanal	8	Velarte	220–260 W wheat flour (Harinas Saiz)	67.5
					380–400 W wheat flour (Harinas Saiz)	25
					Bio T80 flour (Moulin de Colagne)	3.75
					Durum wheat flour (Molí de Picó)	3.75
					Tap water	71.25
					Compressed yeast	1.25
					Table salt	1.8
					Dried sourdough (Sapore Tosca, Puratos)	1.0
					Bread improver (T500, Puratos)	1.0
**G12**	*Pa de la casa* (House bread)	Artisanal	5	Forn d’En Rausell	Whole rye flour (Harinas Santamaría)	60
					380 W wheat flour (Harinas Vicente Bosque)	40
					Tap water	80
					Sourdough	35
					Table salt	1.7
**G13**	*Pa de la casa* (House bread)	Artisanal	4	Forn, Pa i Dolços	220 W wheat flour (Harinas Molí de Picó)	100
					Tap water	70
					Table salt	1.0
					Compressed yeast	1.7

^1^ Bread code. ^2^ Where possible, an English denomination is provided in parentheses. ^3^ The W rating reflects the strength of the flour. Higher W flour has a higher gluten content and allows for longer fermentation. If not indicated, medium-strength flour (W ranging from 180 to 240) is assumed; Tritordeum is a grain developed in Spain by crossing hard wheat with wild barley (https://www.tritordeum.com/tritordeum-bread/, accessed on 4 July 2024); T80 is a high-extraction (0.75 to 0.90% ash) ecologic flour made from stone-ground soft wheat. Main ingredient suppliers are listed in parentheses. Sourdough from artisan bakeries were characterized previously [14]. ^4^ Typically, tap water hardness in the district of the City of Valencia ranges between 400 and 500 mg CaCO_3_/L. Additional information can be found at the SINAC—National Drinking Water Information System—(https://sinac.sanidad.gob.es/CiudadanoWeb/ciudadano/informacionRedes.do, accessed on 25 November 2024). f.b., flour basis.

**Table 2:** Chemical traits of bread dough *.

Bread Dough	SD (%) ^1^	pH	TTA ^2^	Organic Acid (mg/g Dough ^3^)		FQ ^4^
				Lactic Acid	Acetic Acid	
**G1**	20	4.23 ± 0.07 ^h^	8.55 ± 0.07 ^b^	11.2 ± 1.0 ^a^	3.2 ± 0.7 ^a^	2.32
**G2**	20	5.01 ± 0.02 ^def^	2.92 ± 0.04 ^h^	2.5 ± 0.6 ^efg^	0.9 ± 0.4 ^c^	1.85
**G3_F**	20	4.93 ± 0.11 ^ef^	4.57 ± 0.10 ^e^	4.7 ± 0.6 ^bcde^	0.99 ± 0.16 ^c^	3.16
**G3_SDw**	20	3.76 ± 0.08 ^i^	9.8 ± 0.3 ^a^	8.1 ± 2.1 ^ab^	1.0 ± 0.3 ^c^	5.40
**G3_DW**	20	4.66 ± 0.04 ^g^	5.18 ± 0.12 ^d^	5.2 ± 1.1 ^bcde^	0.93 ± 0.13 ^c^	3.73
**G4**	5	5.38 ± 0.05 ^b^	3.04 ± 0.04 ^h^	2.9 ± 0.9 ^efg^	1.11 ± 0.12 ^c^	1.74
**G5_S**	14	4.68 ± 0.03 ^g^	8.53 ± 0.04 ^b^	8.0 ± 1.6 ^ab^	2.13 ± 0.06 ^b^	2.50
**G5_R**	20	4.54 ± 0.03 ^g^	6.35 ± 0.05 ^c^	7.67 ± 1.14 ^abc^	2.5 ± 0.5 ^ab^	2.05
**G6**	30	5.15 ± 0.04 ^cd^	3.4 ± 0.2 ^g^	2.2 ± 0.6 ^efg^	0.7 ± 0.3 ^c^	2.10
**G7**	0	5.58 ± 0.02 ^a^	2.30 ± 0.08 ^i^	0.26 ± 0.04 ^fg^	0.16 ± 0.12 ^c^	1.08
**G9_T**	20	4.87 ± 0.05 ^f^	4.71 ± 0.10 ^e^	4.2 ± 1.1 ^cde^	1.1 ± 0.4 ^c^	2.55
**G9_L**	20	4.53 ± 0.03 ^g^	5.12 ± 0.05 ^d^	4.5 ± 1.2 ^def^	0.8 ± 0.3 ^c^	3.75
**G10**	25	5.09 ± 0.12 ^cde^	4.25 ± 0.05 ^f^	3.3 ± 0.9 ^defg^	0.9 ± 0.4 ^c^	2.44
**G11**	0	5.25 ± 0.02 ^bc^	2.45 ± 0.03 ^i^	1.9 ± 0.5 ^efg^	0.5 ± 0.2 ^c^	2.53
**G12**	35	4.35 ± 0.05 ^h^	6.60 ± 0.14 ^c^	6.7 ± 1.8 ^bcd^	0.6 ± 0.3 ^c^	7.44
**G13**	0	5.58 ± 0.02 ^a^	2.34 ± 0.07 ^i^	0.19 ± 0.02 ^g^	0.4 ± 0.2 ^c^	0.32

* Data represent the mean value (±SD) of at least three independent replicates. Values within a column showing different superscript letters are significantly different (*p* < 0.05) according to Tukey’s test. ^1^ Sourdough (flour basis). ^2^ Total titratable acidity. mL of 0.1 N NaOH needed to adjust the pH of 10 g of bread dough to 8.5. ^3^ Fresh basis. ^4^ FQ, the fermentation quotient is the molar ratio of lactic and acetic acids.

## Data Availability

The original contributions presented in this study are included in the article; further inquiries can be directed to the corresponding author.

## Supplementary Materials

The following supporting information can be downloaded at: https://www.mdpi.com/article/10.3390/foods13233872/s1. Figure S1: Crumb correlation plot; Figure S2: Crust correlation plot; Table S1: Fermentation conditions in the set of bread dough analyzed; Table S2: Main volatile organic compounds (VOCs) in bread crumb; Table S3: Main volatile organic compounds (VOCs) in bread crust.
