# Supplementary material for: Quantitative Assessment of Volatile Profile and Sensory Perception of Artisan Bread Made in the City of Valencia
Source: Foods. 2024 Nov 29;13(23):3872. doi: 10.3390/foods13233872 (PMC11640285; doi:10.3390/foods13233872)
Supplement: Supplementary file 1 [file foods-13-03872-s001.zip › Supplementary material.pdf]

Quantitative assessment of volatile profile and sensory perception of artisan bread made in the city of Valencia

Gemma Sanmartín[a], Isabel E. Sánchez-Adriá[a], Ana Salvador[b], Jose A. Prieto[a], Francisco Estruch[c], and Francisca Randez-Gil[a,]*

[a] *Department of Biotechnology, Instituto de Agroquímica y Tecnología de los Alimentos, Consejo Superior de Investigaciones Científicas, Avda. Agustín Escardino, 7. 46980-Paterna, Valencia, Spain*
[b] *Department of Physical and Sensory Properties of Food and Consumer Science, Instituto de Agroquímica y Tecnología de los Alimentos (IATA-CSIC), Avda. Agustín Escardino, 7. 46980-Paterna, Valencia, Spain*
[b] *Department of Biochemistry and Molecular Biology, Universitat de València, Dr. Moliner 50, 46100-Burjassot, Spain*

Contents:

Table S1. Fermentation conditions in the set of bread dough analyzed
Figure S1. Crumb correlation plot.
Figure S2. Crust correlation plot.

* Correspondence: Francisca Randez-Gil; E-mail: randez@iata.csic.es; Fax: +34 963636301; Tel: +34 963900022

Table S1: Fermentation conditions in the set of bread dough analyzed

Bread	Autolysis (min)	Resting (ºC/min)	Retarding-1 (ºC/h)	Bulk Fermentation (ºC/min)	Retarding-2 (ºC/h)	Pre-proofing (ºC/min)	Proofing (ºC/h)
**G1**	No	25°C/ 20	No	25°C/ 85	5°C/ 8	18°C/ 300	28°C/ 1
**G2**	No	28°C/ 20	No	30°C/ 120	No	No	28°C/ 6
**G3_F**	No	28°C/ 30	7°C/ 10	No	No	28°C/ 120	7°C/ 8
**G3_SDw**	No	28°C/ 20	7°C/ 10	28°C/ 20	No	28°C/ 60	15°C/ 6
**G3_DW**	No	28°C/ 30	No	No	No	No	12°C/ 16
**G4**	No	28°C/ 20	No	No	No	No	15°C/ 4
**G5-S**	No	30°C/ 20	No	30°C/ 70	No	No	24°C/ 2
**G5-R**	No	30°C/ 20	No	30°C/ 70	No	No	24°C/ 2
**G6**	45	26°C/ 30	No	26°C/ 210	No	26°C/ 20	7°C/ 18
**G7**	No	26°C/ 20	4°C/ 18	No	No	No	25°C/ 0.5
**G9-T**	No	28°C/ 20	No	28°C/ 65	No	28°C/ 40	8°C/ 12
**G9-L**	40	25°C/ 30	No	25°C/ 180	No	25°C/ 30	8°C/ 12
**G10**	30	25°C/ 20	No	25°C/ 100	4°C/ 18	25°C/ 55	8°C/ 18
**G11**	90	30°C/ 20	No	30°C/ 70	2°C/ 21	No	32°C/ 3
**G12**	No	25°C/ 70	6°C/ 15	23°C/ 210	No	No	23°C/ 4.5
**G13**	90	25°C/ 20	No	25°C/ 70	No	No	25°C/ 2

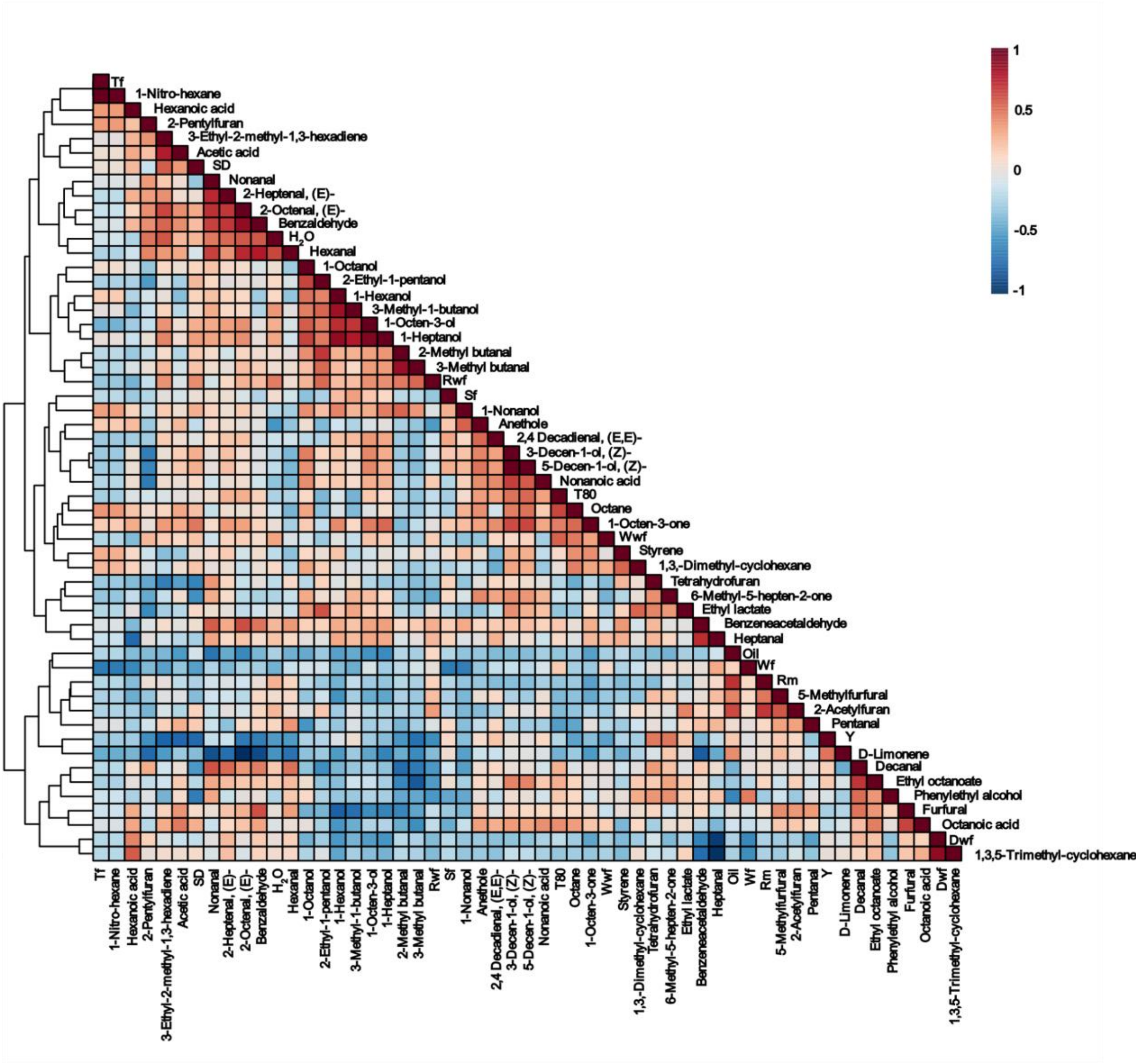

Figure S1. Crumb correlation plot. Pearson's correlation between pairs of main ingredients and 43 VOCs identified (Table S1) in crumb samples from the set of artisan breads analysed (Table 1). Volatile compounds exclusively detected in control industrial bread (Table S1) were not considered. Abbreviations for ingredients are as follows: Wheat flour (Wf); Whole wheat flour (Wwf); T80 flour (T80); Water (H2O); Sourdough (SD); Yeast (Y); Whole rye flour (Wrf); Roasted malt (Rm); Durum wheat flour (Dwf); Tritordeum flour (Tf); Spelt flour (Sf). The Pearson correlation index is indicated by the scale bar, with 1 denoting perfect positive correlation (dark red) and -1 denoting perfect negative correlation (dark blue).

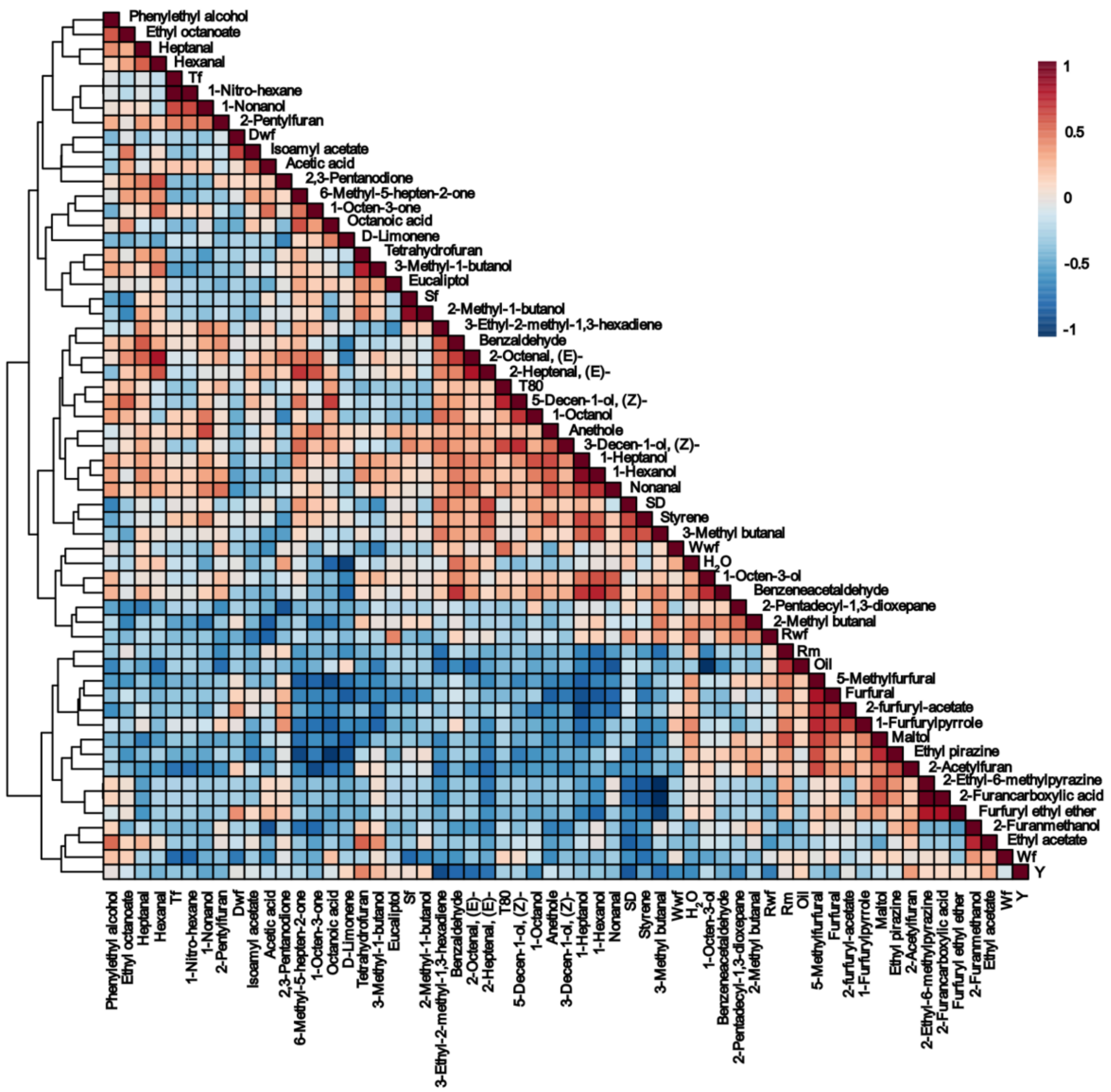

Figure S2. Crust correlation plot. Pearson's correlation between pairs of main ingredients and 47 VOCs identified (Table S2) in crust samples of the set of artisan breads analysed (Table 1). Volatile compounds exclusively detected in control industrial bread (Table S2) were not considered. Abbreviations for ingredients are as follows: Wheat flour (Wf); Whole wheat flour (Wwf); T80 flour (T80); Water (H_2O); Sourdough (SD); Yeast (Y); Whole rye flour (Wrf); Roasted malt (Rm); Durum wheat flour (Dwf); Tritordeum flour (Tf); Spelt flour (Sf). The Pearson correlation index is indicated by the scale bar, with 1 denoting perfect positive correlation (dark red) and -1 denoting perfect negative correlation (dark blue).
